# α-Galactosidase a Deficiency in Fabry Disease Leads to Extensive Dysregulated Cellular Signaling Pathways in Human Podocytes

**DOI:** 10.3390/ijms222111339

**Published:** 2021-10-20

**Authors:** Ulrich Jehn, Samet Bayraktar, Solvey Pollmann, Veerle Van Marck, Thomas Weide, Hermann Pavenstädt, Eva Brand, Malte Lenders

**Affiliations:** 1 Department of Medicine D, Division of General Internal and Emergency Medicine, Nephrology, and Rheumatology, University Hospital Münster, 48149 Münster, Germany; Ulrich.Jehn@ukmuenster.de (U.J.); samet.bayraktar@outlook.de (S.B.); weidet@uni-muenster.de (T.W.); Hermann.Pavenstaedt@ukmuenster.de (H.P.); 2 Internal Medicine D, Department of Nephrology, Hypertension and Rheumatology, Interdisciplinary Fabry Center (IFAZ), University Hospital Münster, 48149 Münster, Germany; Solvey.Pollmann@ukmuenster.de (S.P.); Eva.Brand@ukmuenster.de (E.B.); 3 Gerhard-Domagk-Institute of Pathology, University Hospital Münster, 48149 Münster, Germany; vanmarck.veerle@gmail.com

**Keywords:** Fabry disease, podocytes, α-galactosidase A-deficiency, proteome analysis, sphingolipids

## Abstract

Fabry disease (FD) is caused by mutations in the α-galactosidase A (*GLA*) gene encoding the lysosomal AGAL enzyme. Loss of enzymatic AGAL activity and cellular accumulation of sphingolipids (mainly globotriaosylcermide) may lead to podocyturia and renal loss of function with increased cardiovascular morbidity and mortality in affected patients. To identify dysregulated cellular pathways in FD, we established a stable AGAL-deficient podocyte cell line to perform a comprehensive proteome analysis. Imbalanced protein expression and function were analyzed in additional FD cell lines including endothelial, epithelial kidney, patient-derived urinary cells and kidney biopsies. AGAL-deficient podocytes showed dysregulated proteins involved in thermogenesis, lysosomal trafficking and function, metabolic activity, cell-cell interactions and cell cycle. Proteins associated with neurological diseases were upregulated in AGAL-deficient podocytes. Rescues with inducible AGAL expression only partially normalized protein expression. A disturbed protein expression was confirmed in endothelial, epithelial and patient-specific cells, pointing toward fundamental pathway disturbances rather than to cell type-specific alterations in FD. We conclude that a loss of AGAL function results in profound changes of cellular pathways, which are ubiquitously in different cell types. Due to these profound alterations, current approved FD-specific therapies may not be sufficient to completely reverse all dysregulated pathways.

## 1. Introduction

Fabry disease (FD) is an X-linked progressive multisystemic disorder resulting from lysosomal enzyme α-galactosidase A (AGAL) deficiency. The loss of enzymatic AGAL activity leads to a progressive lysosomal accumulation of mainly globotriaosylceramide (Gb_3_), resulting in early stroke, renal and cardiac failure, and malignant arrhythmia significantly limiting life expectancy in affected patients by up to 15 years [1]. Renal failure includes progressive loss of glomerular filtration and increasing albuminuria. Gb_3_ accumulation in podocytes correlates with progressive podocyte loss [2]. Podocyturia is associated with the clinical severity of Fabry nephropathy and thus of prognostic importance [3,4,5]. On the cellular level, preliminary data suggest that the accumulation of Gb_3_ leads to a disturbance of molecular pathways within affected cells including disturbed autophagy and inflammation [6]. In addition, soluble globotriaosylsphingosine (lyso-Gb_3_) activates podocyte Notch1-signaling in cell culture, which was confirmed by kidney biopsies from FD patients [7].

FD is treatable with recombinant enzyme replacement therapy (ERT) since 2001 and chaperone therapy (Migalastat, Amicus) since May 2016. FD-specific treatment may result in a Gb_3_ clearance of podocytes in vitro [8] and in vivo [9]. However, although short-term treatment over 3 days with ERT confirms a reduction of Gb_3_ content in podocytes in vitro, disturbed molecular pathways such as autophagy, mTOR/AKT signaling and pro-fibrotic signaling are not positively affected [8]. A major limitation of these cell-based studies is that AGAL activity in podocytes was only transiently and thus not fully inhibited by shRNA [8], and that wild-type podocytes were treated with pathologic lyso-Gb_3_ concentrations [7] only, not representing a real FD-typical pathological situation, based on genomic alterations (i.e., missense and nonsense mutations) in vivo. To overcome this drawback, we generated a stable AGAL-knockout FD podocyte cell line using an established CRISPR/Cas9-mediated approach [10,11] and performed comprehensive proteomic analyses to identify potential altered pathways. In addition, to mimic novel gene correction approaches (i.e., gene therapy), we performed an inducible AGLA knock-in in FD podocytes. Finally, dysregulated protein expression and function was analyzed in additional FD cell lines, including immortalized endothelial cells, epithelial kidney cells, patient-derived urinary cells and a representative kidney biopsy.

## 2. Results

**Figure 1:**
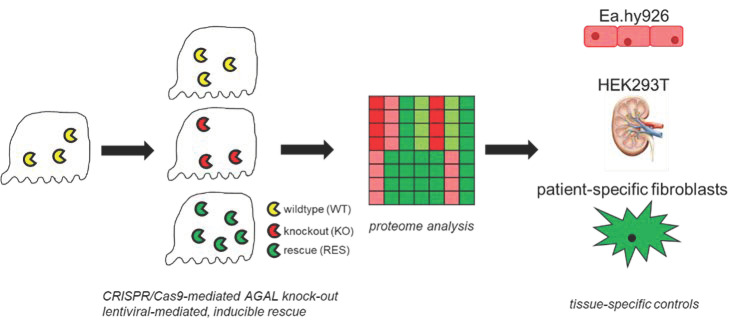
Overview of the study procedure. CRISPR/Cas9-mediated AGAL (α-galactosidase A) knockout (KO) podocytes were compared to wild-type (WT) and lentiviral-mediated rescued (RES) cells via proteome analysis. Expression of dysregulated proteins of interest was further analyzed in endothelial (Ea.hy926), kidney epithelial (HEK293T) cells and patient-specific fibroblasts.

Progressive loss of renal function including decreasing eGFR and increasing albuminuria over time belongs to the main manifestations in affected patients with FD. Associated with these processes, several studies showed a progressive loss of podocytes (podocyturia) in affected patients [4,5,12,13,14]. FD in vitro podocyte models [6] suggested a dysregulated protein expression for several important cellular pathways. The aim of this study was to generate stable AGAL-deficient human podocytes to identify differences in protein expression between wild-type and representative FD podocytes in more detail (Figure 1). A further aim was to analyze the effect of genetically rescued AGAL podocytes and the subsequent expression of candidate genes in other tissues and patient- and mutation-specific cell lines (Figure 1).

**Figure 2:**
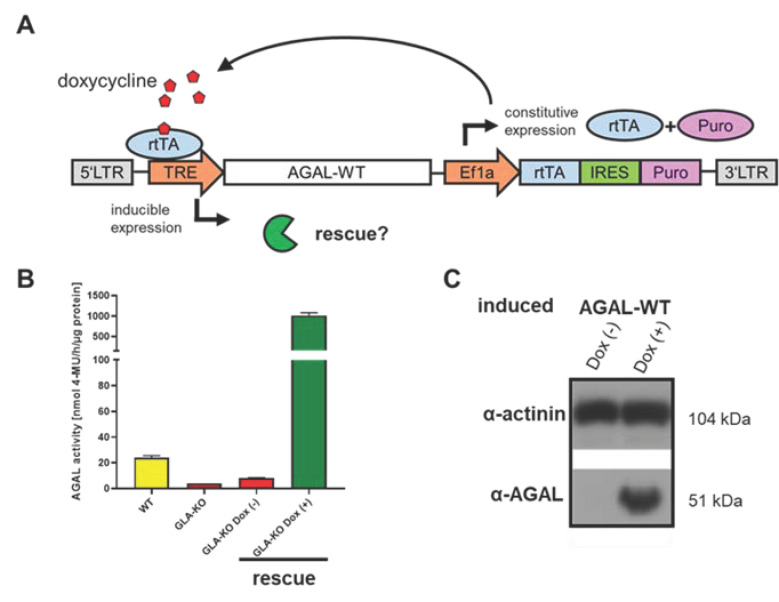
Generation of an inducible AGAL and mutant rescue in Fabry podocytes. (**A**) Schematic overview of the doxycycline inducible pINDUCER construct (tet on—tet off) driving wild-type AGAL expression after stable lentiviral-mediated genomic insertion. (**B**) AGAL activities in wild-type, AGAL-deficient and lentiviral-mediated transfected podocytes with and without doxycycline induction. (**C**) Western blot analysis of AGAL expression in lentiviral-mediated rescued wild-type doxycycline-induced podocytes. AGAL: α-galactosidase A, Dox (-): without doxycycline, Dox (+): with doxycycline, Ef1a: elongation factor 1 alpha, IRES: internal ribosomal entry site, LTR: long terminal repeat, Puro: puromycin resistance, rtTA: reverse tetracycline-controlled transactivator, TRE: tetracycline response element, WT: wild-type.

**Figure A1:**
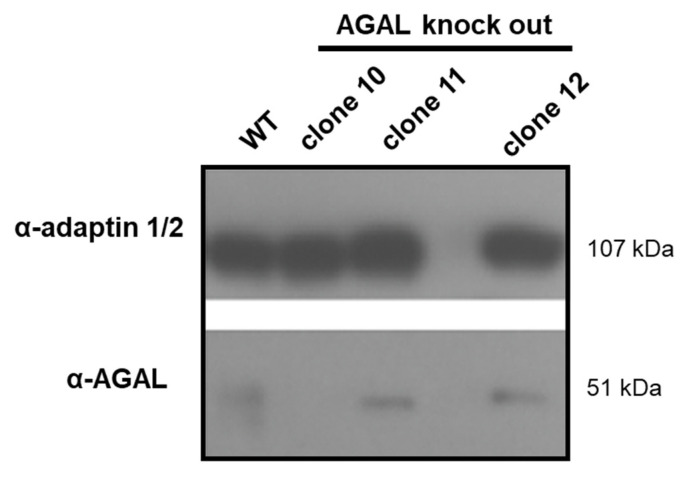
Western blot analysis of AGAL expression in wild-type podocytes (AB8) and CRISPR/Cas9-mediated AGAL-deficient clones. AGAL: α-galactosidase A, WT: wild-type.

To generate a stable CRSPR/Cas9-mediated FD podocyte cell line, a previously established gRNA, binding at cDNA position c.481 was used [10,11]. Single cell separation identified several clones lacking endogenous AGAL activity (Figure A1, Appendix A). Clone 10, with a deletion of four base pairs leading to a lack of asparagine acid at amino acid position 161 followed by a frameshift with subsequent termination, was used for further experiments (c.481del4). This nonsense mutation resulted in an endogenous enzymatic AGAL activity < 10% of wild-type activity (Figure 2B) and was no longer detectable by Western blot analyses (Figure A1, Appendix A). Next, inducible AGAL-rescue podocytes were generated by lentiviral-mediated stable genomic integration with this AGAL-deficient podocyte clone using wild-type AGAL (Figure 2A). Doxycycline induction led to an increased expression of wild-type AGAL resulting in high enzymatic AGAL activity and quantity (Figure 2B,C).

### 2.1. Proteome Analyses

#### 2.1.1. Comparison of Replicates and Groups for LC-MS/MS Reproducibility and Quality

**Figure 3:**
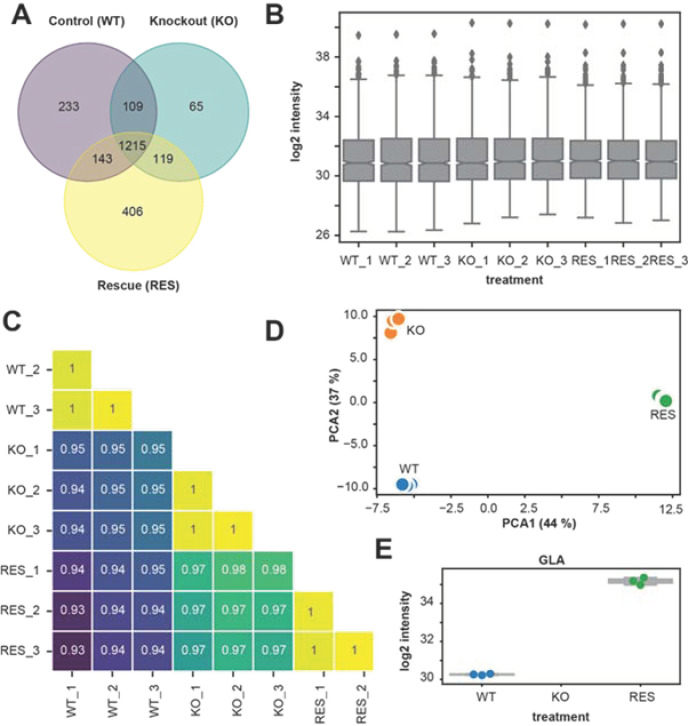
Comparison of replicates and groups for LC-MS/MS reproducibility. (**A**) Venn diagram showing the distribution of identified proteins in different groups (wild-type/control (WT)), knockout (KO), and rescue (RES). (**B**) Boxplots showing the label-free quantification (LFQ) intensities (log2) of all biological replicates. (**C**) Correlation map of biological replicates for all groups. It shows a high Pearson correlation coefficient of LFQ intensities between replicates of the same group (> 0.99). (**D**) Principal component analysis (PCA) of biological replicates for all groups. Visualization shows strong clustering between replicates of the same group. (**E**) log2 intensity of AGAL/GLA-protein for each group and replicates, confirming knockout and rescue.

LC-MS/MS spectral analysis was used to identified proteins and label-free quantification (LFQ) intensities to compare different groups: (1) wild-type (WT), (2) AGAL-deficient (knockout (KO)), (3) doxycycline-inducible rescue (RES). Based on LFQ intensities and LC-MS/MS spectral analysis, we identified 2290 proteins in total. A protein was declared as present, if at least the LFQ intensity among the triplicate of one group was non-zero. A Venn diagram (Figure 3A) illustrates shared and unique identified proteins in each group. Among 2290 of all identified proteins, 1700 proteins (74.2%) in wild-type, 1508 (65.8%) in KO, and 1883 (82.2%) in the rescue were identified. A total of 1215 shared proteins (53.1%) were present in all replicates and groups. The numbers of unique proteins were 233 (13.7%) in wild-type, 65 (4.3%) in KO and 406 (21.6%) in the rescue. Figure 3B summarizes log2 LFQ intensities of all replicates for each group as boxplot, showing comparable values across replicates, confirming a high consistency of the LC-MS/MS measurement. Pearson correlation coefficient analysis of LFQ intensities showed a high correlation coefficient between biological replicates (R2 > 0.99), indicating low intragroup variabilities compared to the intergroup variabilities, too (Figure 3C). In addition, a principal component analysis of LFQ intensities of the 1215 common and shared proteins confirmed a high clustering between replicates for each group, also indicating consistency and low variability of the biological replicates (Figure 3D). The analysis of AGAL expression confirmed the knockout in all replicates of the KO group (below the limit of detection) in comparison to the wild-type (mean LFQ intensity = 30.2), whereas the AGAL protein expression in the rescue (mean LFQ intensity = 35.2) was on average ~32-fold higher than in the wild-type (Figure 3E). A comprehensive overview of all dysregulated proteins is provided in Supplementary Table S1.

#### 2.1.2. Regulated Proteins in Pairwise Comparison

**Figure 4:**
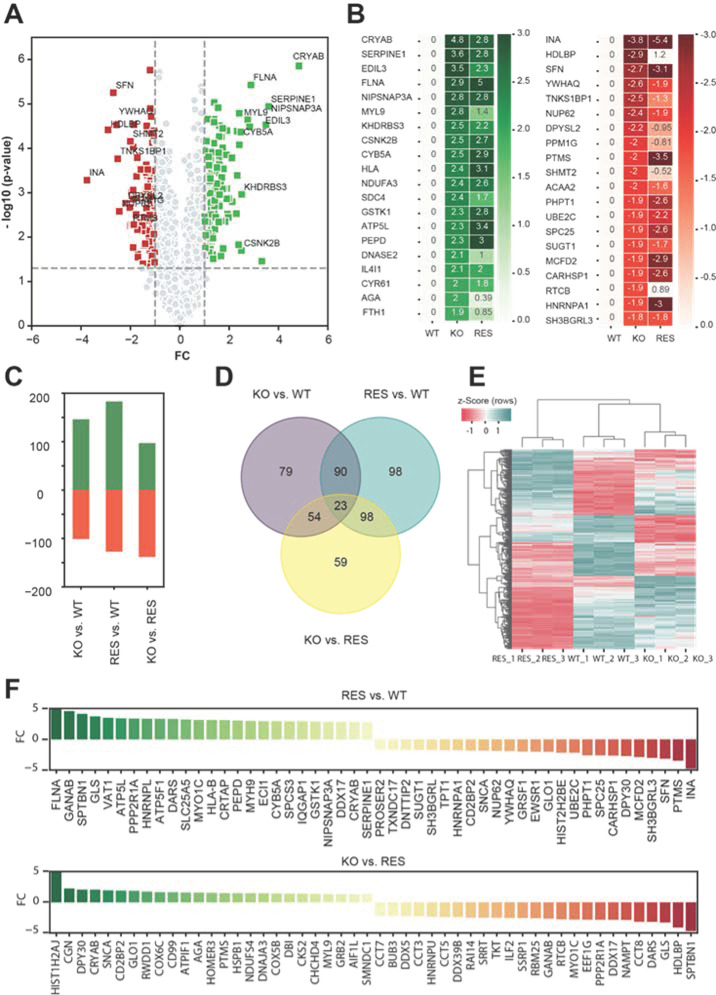
Overview of differentially regulated proteins in pairwise comparison. (**A**) Volcano plot illustrates the differentially regulated proteins (related to gene names) in the reference comparison group between knockout (KO) and wild-type (WT). *X*-axis represents the log2-fold changes (FC) of LFQ intensities and *y*-axis –log10 (*p* values). Up- and downregulated proteins in KO are labelled in green and red squares. (**B**) Heat map showing the average log2 LFQ intensities of top 20 up- and downregulated proteins from reference (KO vs. WT) in comparison to rescue (RES). (**C**) Bar charts representing the number of significantly regulated proteins in pairwise comparison. (**D**) Venn diagram illustrates the distributions of the significantly altered proteins from the pairwise comparisons. (**E**) Hierarchical clustering of the significantly regulated proteins from the pairwise comparisons. Single LFQ intensities of proteins are clustered in rows and columns using centroid linkage method and Euclidian distance measurement technique. Z-Score was used to normalize the data. Upregulated and downregulated proteins are represented with red and green colors. (**F**) The expression levels of top 25 significantly (trimmed log2 FC of 5, *p* value < 0.05) up- and downregulated proteins in different pairwise comparison groups: RES vs. WT, KO vs. RES.

Next, log2 LFQ intensities were used to compare two groups and calculate the differentially expressed proteins (log2-fold changes) in three pairwise comparison groups: wild-type vs. KO, wild-type vs. rescue, and KO vs. rescue. Figure 4A shows a Volcano plot, representing statistical significance and log2-fold changes between wild-type and KO, which is the reference comparison group. The top 20 up- and downregulated proteins are presented in Figure 4B. Among these proteins, the rescue had a positive effect on myosin regulatory light polypeptide 9 (MYL9), syndecan 4 (SDC4), deoxyribonuclease II (DNASE2), aspartylglucosaminidase (AGA), ferritin heavy chain 1 (FTH1), tankyrase 1 binding protein 1 (TNKS1BP1), dihydropyrimidinase-like 2 (DPYSL2), protein phosphatase 1G (PPM1G), serine hydroxymethyltransferase 2 (SHMT2), and RNA 2’’,3’-cyclic phosphate and 5’-OH ligase (RTCB) protein expression (Figure 4B). However, among the significantly dysregulated proteins, alpha-crystallin B chain (CRYAB), filamin A (FLNA), EGF-like repeats and discoidin domains 3 (EDIL3), and plasminogen activator inhibitor type 1 (SERPINE1) were still highly upregulated and alpha-internexin (INA), stratifin (SFN), nucleoporin p62 (NUP62), parathymosin (PTMS) and Heterogeneous nuclear ribonucleoprotein A1 (HNRNPA1) were highly downregulated in KO as well as in rescued cells (Figure 4B).

An overview of all dysregulated proteins (up- and downregulated) between the tree comparison groups is provided in Figure 4C–E. A cluster map shows all significantly dysregulated proteins in all three pairwise comparisons (Figure 4E). Interestingly, the KO was closer to the wild-type than the rescue, potentially indicating an absent rescue effect as well as a negative effect of induced AGAL expression in KO cells. Figure 4F illustrates the top 25 up- and downregulated proteins (*p* < 0.05) and their expression levels (log2-fold changes of LFQ intensities) for rescue versus wild-type cells and KO versus rescued cells.

#### 2.1.3. Gene-Set Enrichment Analysis

**Figure 5:**
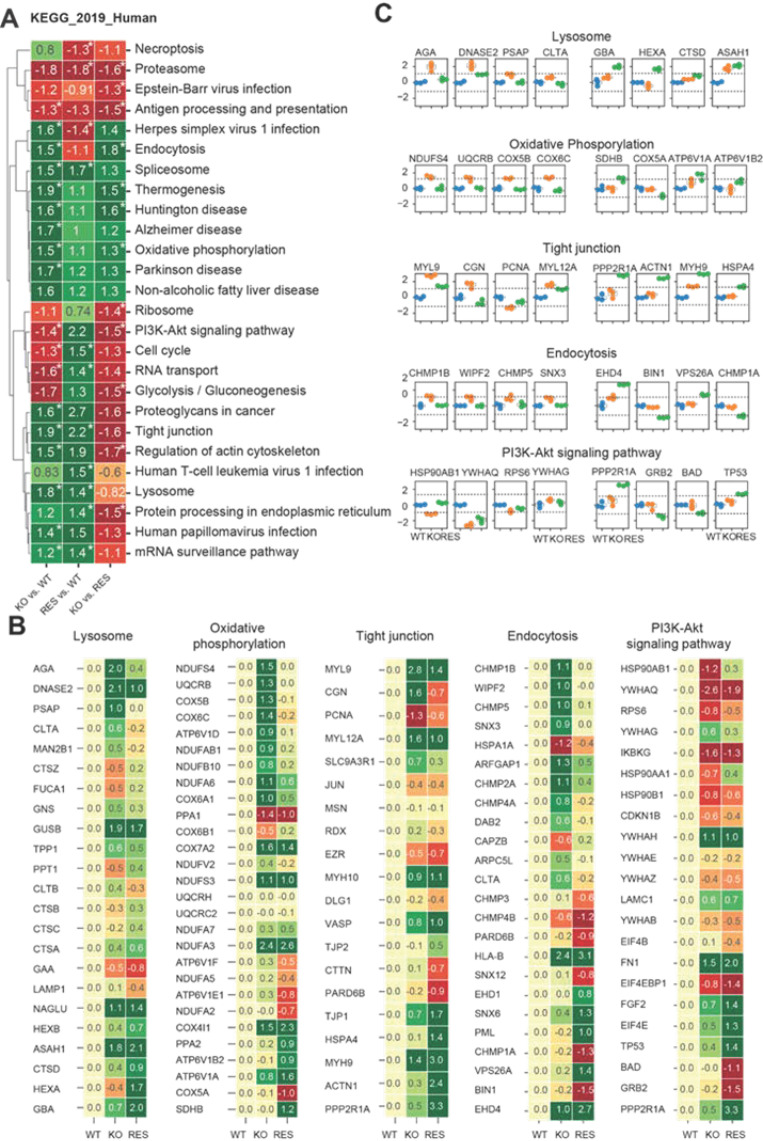
Gene set enrichment analysis using KEGG library to analyze the effects of AGAL rescue. (**A**) Hierarchical clustering of identified up- and downregulated pathways illustrate normalized enrichment scores (nes) from GSEA of the pairwise comparison (KO vs. WT, RES vs. WT, KO vs. RES). An asterisk marks significant changes. (*p* value = 0.05, fdr = 0.25). (**B**) Heat maps representing N-fold LFQ mean values of most significant misregulated proteins in 5 representative pathways. For each protein, the log2 mean values were subtracted from WT. Candidates on the top of the heat map represents the strongest rescue and lower the opposite effect. (**C**) Expression of representative proteins from different pathways. Left boxplots represent proteins with strongest rescue effect and right boxplots the opposite effect for KO. blue: WT, orange: KO, green: RES.

**Figure A2:**
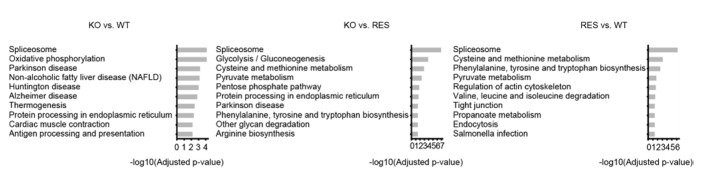
Gene ontology analysis of significantly regulated proteins. Bar charts representing the 10 top enriched terms of significantly regulated proteins in pairwise comparison of the groups (KO vs. WT, KO vs. RES and RES vs. WT).

We additionally performed a gene set enrichment analysis (GSEA) (Figure 5A) and gene ontology (GO) analysis (Figure A2, Appendix A) using KEGG as a library to identify significantly altered pathways between wild-type, KO and rescued cells. The aim was to detect enrichment terms (*p* < 0.05, fdr < 0.25) for human diseases and cellular components resulting from the AGAL knockout and to analyze the effects of AGAL rescue (Supplementary Tables S2 and S3). The normalized enrichment score (nes) was used to compare the pairwise gene set enrichment analysis of the groups (KO vs. WT, RES vs. WT and KO vs. RES). Based on the enrichment analysis, we identified in AGAL-deficient podocytes several terms/pathways, which were linked to cellular components and processes as dysregulated (Figure 5A). In detail, in AGAL-deficient podocytes, we identified proteins associated within the following terms as significantly upregulated: endocytosis, spliceosome, thermogenesis, Huntington’s disease, Alzheimer’s disease, oxidative phosphorylation, non-alcoholic fatty liver disease, Parkinson’s disease, proteoglycans in cancer, tight junction, regulation of actin skeleton, lysosome, human papillomavirus infection, and mRNA surveillance pathway. Vice versa, proteins associated with antigen processing and presentation, PI3K-Akt signaling pathway, cell signaling, and RNA transport were downregulated (Figure 5A). By comparing KO with wild-type and KO with rescued cells to identify effects of an AGAL rescue, we identified the following terms and pathways as normalized after rescue: endocytosis, thermogenesis, Huntington’s disease, oxidative phosphorylation, and PI3K-Akt signaling pathway (Figure 5A). No effects of an AGAL rescue were observed for the spliceosome, tight junctions, mRNA surveillance pathway and importantly also for the lysosome (Figure 5A). Of note, the rescue itself led to an increased expression of proteins involved in protein processing within the endoplasmic reticulum, probably due to the increased expression of AGAL. Next, we analyzed the effects of AGAL rescue on protein expression within the terms lysosome, oxidative phosphorylation, tight junctions, endocytosis, and PI3K-Akt signaling pathways in more detail (Figure 5B,C). A beneficial effect of AGAL rescue on protein expression was most dominant for AGA, DNASE2, prosaposin (PSAP) and clathrin light chain A (CLTA) for lysosome, NADH-ubiquinone oxidoreductase 18 kDa subunit (NDUFS4), ubiquinol-cytochrome c reductase binding protein (UQCRB), cytochrome c oxidase subunit 5B (COX5B) and Cytochrome c oxidase subunit 6C (COX6C) for oxidative phosphorylation, myosin regulatory light polypeptide 9 (MYL9), cingulin (CGN), proliferating cell nuclear antigen (PCNA), myosin regulatory light chain 12A (MYL12A) for tight junction, charged multivesicular body protein 1b (CHMP1B), WAS/WASL-interacting protein family member 2 (WIPF2), charged multivesicular body protein 5 (CHMP5) and sorting nexin-3 (SNX3) for endocytosis and heat shock protein HSP 90-beta (HSP90AB1), 14-3-3 protein theta (YWHAQ) and ribosomal protein s6 (RPS6) for PI3K-Akt signaling pathways (Figure 5C).

### 2.2. Expression of GOIs and Functional Analyses of Involved Pathways in Other FD Cell Lines and Patient-Specific Fibroblasts

**Figure 6:**
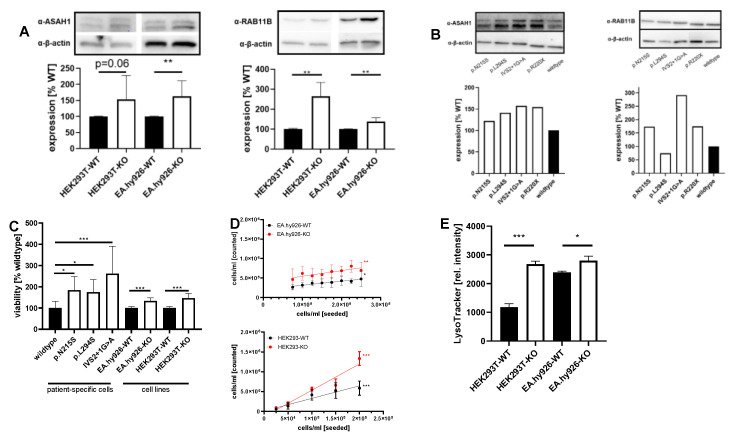
Expression of GOIs and functional analyses of involved pathways in other FD cell lines and patient-specific fibroblasts. (**A**) ASAH1 and RAB11B expression in kidney epithelial HEK293T, endothelial EA.hy926 cells and (**B**) in patient-derived fibroblast cells compared to a healthy control (wild-type). (**C**) Increased cell viability and (**D**) proliferation in AGAL-deficient cells. (**E**) Increased lysosomal volume in AGAL-deficient EA.hy926 and HEK293T cells. * *p* < 0.05, ** *p* < 0.01, *** *p* < 0.001.

**Figure 7:**
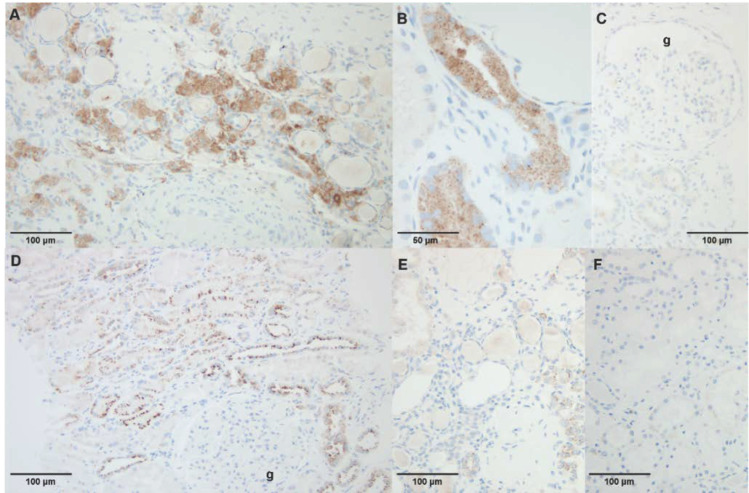
ASAH1-stainings in kidney biopsy specimen. (**A**–**C**) specimen from a male FD patient (34 years of age) with a classical phenotype with progressive loss of eGFR and macroalbuminuria showing increased ASAH1 expression in the tubular epithelial cells, but not in the glomeruli (g). (**D**) Male non-FD control patient (50 years of age) with chronic renal insufficiency due to hypertensive nephropathy. (**E**,**F**) Male non-FD control patient (51 years of age) with normal renal tissue adjacent to a primary kidney tumor (with primary antibody, (**E**), and negative control without primary antibody, (**F**).

**Figure A3:**
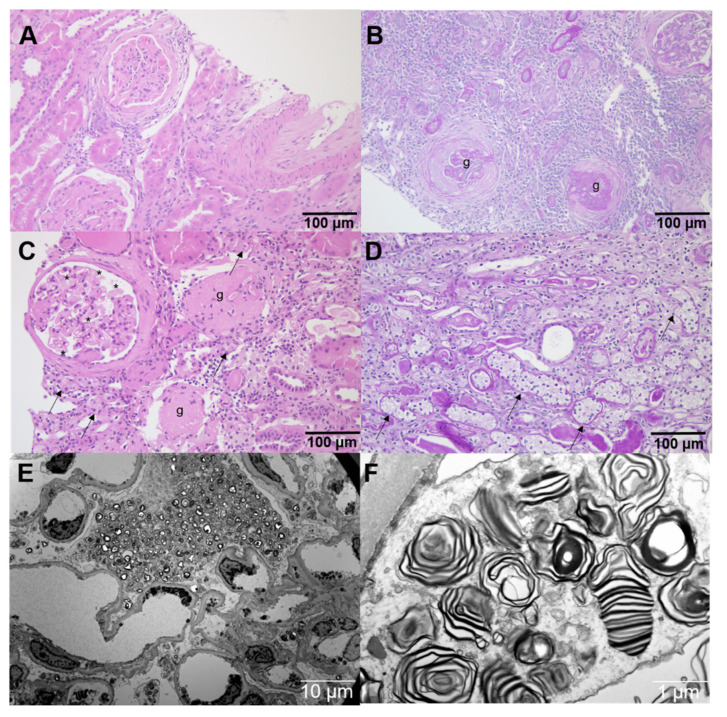
Additional histology and electron microscopy. HE- and PAS-stained paraffin sections from (**A**,**B**) a non-FD control patient with hypertensive nephropathy, showing arteriosclerosis and chronic parenchymal damage with glomerulosclerosis (g), interstitial fibrosis and tubular atrophy, as well as (**C**,**D**) from a classical FD patient, showing vacuolated glomerular (*) and tubular (arrows) epithelial cells, in addition to signs of disease progression with global glomerulosclerosis (g). Electron microscopy (**E**,**F**) from the classical FD patient at ERT-naïve status showing FD-typical electron-dense multilamellar inclusion bodies (zebra bodies), which were pivotal for the diagnosis.

To assess if dysregulated genes and pathways identified in FD podocytes might also be affected in other AGAL-deficient cells, we performed additional expression-based and functional experiments with identified candidates in AGAL-deficient endothelial EA.hy926 [11], kidney epithelial HEK293T [10] as well as FD patient-derived urinary fibroblast-like cells [10]. Western blot analyses revealed for both FD cell lines (EA.hy926 and HEK293T) an increased expression of N-acylsphingosine amidohydrolase 1 (ASAH1, an acid ceramidase that converts Gb_3_ in hydrophilic and soluble lyso-Gb_3_, an important marker of disease burden in FD) and Ras-related protein Rab-11B (RAB11B, recycling of proteins from the endosomes to the plasma membrane), too (Figure 6A). ASAH1 and RAB11B expression were also analyzed in FD patient-derived fibroblast-like cells representing different FD-specific *GLA* mutations. Western blot analyses showed an increased expression of both proteins in these cells compared to cells from a healthy control patient (Figure 6B). To further substantiate our findings, we performed ASAH1 immunohistochemical staining in kidney specimens. For this purpose, we randomly selected a control renal biopsy from a patient suffering hypertensive nephropathy (Figure A3A,B), normal renal tissue adjacent to a primary kidney tumor, as well as a kidney biopsy from a classical male FD patient (GLA mutation: c.723dupT) with FD-typical multi-lamellar cellular inclusions at ERT-naïve status (Figure A3E,F, Appendix A) and progressive kidney disease despite ERT (Figure A3C,D, Appendix A). The control kidney tissue showed distinct granular ASAH1 immunoreactivity in the cytoplasm of a subset of the tubular epithelial cells (Figure 7D,E). A similar expression pattern was observed in the kidney biopsy from the FD patient, but ASAH1 immunoreactivity was clearly enhanced (Figure 7A,B). Of note, we did not detect any significant glomerular ASAH1 expression, neither in the FD (Figure 7C), nor in the non-FD patient (Figure 7D).

Proteomic data additionally demonstrated disturbances for cell cycle and replication (pathways in cancer) in AGAL-deficient podocytes. Cell viability and proliferation assays with FD cell lines confirmed these data, in that viability rates were increased in AGAL-deficient EA.hy926 and HEK293T cells (~1.34-fold and 1.46-fold, respectively, both *p* = 0.001) as well as FD-patient derived fibroblast-like cells (Figure 6C), resulting in 1.4-fold higher proliferation rates in AGAL-deficient EA.hy926 and 2.08-fold higher rates in HEK293T cells (Figure 6D). These data point to higher metabolic rates, which could also lead to mitochondrial stress due to an increased NADH and ATP turnover. Intracellular lysosome staining with LysoTracker dyes revealed higher intensities and thus increased lysosomal volumes in AGAL-deficient cells (Figure 6D).

## 3. Discussion

Glomerular podocytes are unique cells with complex foot processes covering the outer layer of the glomerular basement membrane and are the principal cells comprising filtration barriers of glomerular capillaries. In FD, podocytes accumulate Gb_3_ over time, leading to a loss of function and progressive podocyturia. Progressive loss of renal function including decreasing GFR and increasing albuminuria is one of the major manifestations and risk predictors for mortality in FD. To analyze the effect of enzymatic AGAL deficiency on protein expression in these specialized cells, we generated stable AGAL-deficient podocytes and performed a comprehensive proteome analysis to identify dysregulated pathways. In short, our results were: (1) AGAL-deficient podocytes showed dysregulated proteins involved in thermogenesis, lysosomal trafficking and function, metabolic activity, cell-cell interactions and cell cycle. (2) Proteins related to neurologic diseases were upregulated in AGAL-deficient podocytes, too. (3) Rescue experiments with inducible AGAL expression only partially rescued observed effects in AGAL-deficient podocytes. (4) A dysregulation of representative proteins was confirmed in endothelial, epithelial and patient-specific cells, pointing toward fundamental pathway disturbances rather than to cell type-specific alterations in FD.

A recent study demonstrated major metabolic alterations in AGAL-deficient tubular cells, severely affecting renal energy metabolism and underlining their role in chronic kidney disease (CKD) progression [15]. In addition, in urine-derived primary cells of FD patients, the lack of AGAL and constant accumulation of Gb_3_ leads to a dysregulation of molecular pathways including disturbed autophagy and inflammation [6]. Furthermore, lyso-Gb_3_ activates Notch1-signaling in podocytes in cell culture [7]. In the same work, the authors also confirmed increased Notch 1 expression and thus increased inflammation in kidney biopsies from FD patients [7]. The pro-inflammatory state was further confirmed in that tubular epithelial cells in biopsies from FD patients express TGF-ß1 and peritubular interstitial and glomeruli cells are positive for further myofibroblasts markers [16]. Our data confirmed previously identified dysregulated pathways and additionally identified the most affected involved proteins. In detail, we confirmed proteins associated with endocytosis, spliceosome, thermogenesis, Huntington’s disease, Alzheimer’s disease, oxidative phosphorylation, non-alcoholic fatty liver disease, Parkinson’s disease, proteoglycans in cancer, tight junction, regulation of actin skeleton, mRNA surveillance pathway, antigen processing and presentation, PI3K-Akt signaling pathway, cell signaling, RNA transport and of course the lysosome as dysregulated in AGAL-deficient podocytes.

### 3.1. Dysfunction of the Lysosomal Pathways

**Figure 8:**
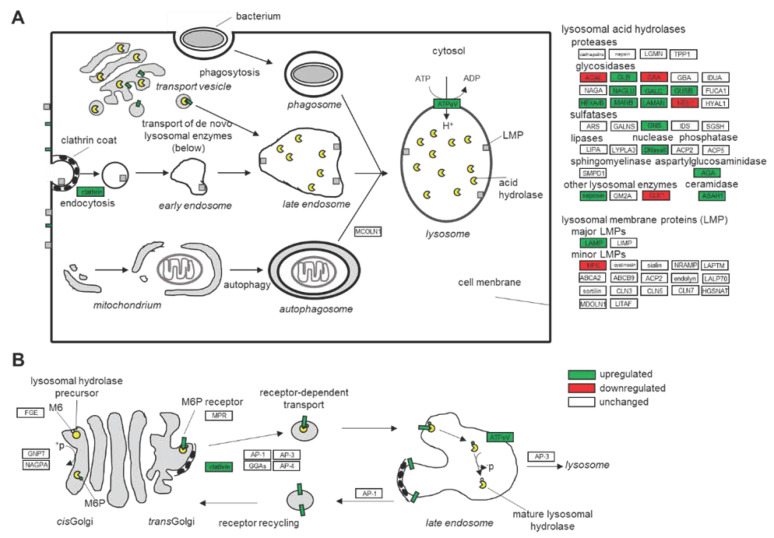
Overview of mainly dysregulated proteins in AGAL-deficient podocytes compared to wild-type. (**A**) Overall lysosomal-associated proteins. (**B**) Translocation between the Golgi com-plex and the late endosome.

Since FD is a lysosomal storage disease, we focused on dysregulated proteins involved in lysosomal trafficking and function. The lack of functional AGAL and resulting lysosomal Gb_3_ accumulation leads to an increased expression of other lysosomal hydrolases such as ASAH1, N-acetylglucosaminidase (NAGLU), β-glucuronidase (GUSB), hexosaminidase A and B (HEXA, HEXB), alpha-mannosidase (MANB) and lysosomal alpha-mannosidase (LAMAN) (Figure 8). Gb_3_ accumulation further seems to increase pH in lysosomes, requiring an increased ATPeV expression for re-acidification. This process requires high amounts of ATP, which in turn might increase metabolic stress, potentially explaining the dysregulated proteins involved in oxidative phosphorylation as well as mitochondrial stress, which were also previously identified [15]. Furthermore, increased clathrin expression points toward increased endocytosis as well as increased protein synthesis followed by intracellular trafficking toward lysosomes. Increased protein expression leading to ER stress is also indicated by increased alpha-crystallin B chain (CRYAB) expression, which is part of the small heat shock protein family and functions as molecular chaperone that primarily binds misfolded proteins to prevent protein aggregation.

### 3.2. Comparison of the Results to Other Chronic Kidney Diseases and to Other FD-Typical Manifestations

Comprehensive proteome expression data for FD are yet limited. However, the cellular mechanisms responsible for kidney damage in FD seem to share similarities with other CKD. Proteome analysis of purified podocyte fractions from focal segmental glomerulosclerosis (FSGS) mouse models showed an early stress response that includes perturbations of metabolic, mechanical, and in proteostasis-involved proteins [17]. Dysregulated protein expression leading, i.e., to a dysfunction of energy transduction in these specialized cells may underlie the podocyte injury associated with numerous glomerular diseases [18].

Human podocytes mimicking diabetic kidney disease by bradykinin treatment showed an inhibition of cell death-associated pathways, engagement of cytoskeletal elements and activation of inflammatory pathways [19]. Inflammatory proteins that were identified to be induced were cyclooxygenases (COX) proteins [19], which are involved in inflammation and were also increased in our AGAL-deficient podocytes. Dysregulated proteins involved in the formation of tight junctions might further explain FD-typical podocyte foot-processes effacement and eventually podocyturia due to decreased cell-cell connections.

Dysregulated genes involved in thermogenesis might explain why classical FD patients present with an inability to sweat, reduced physical exercise capacity and suffer from fatigue due to an increased cellular metabolism and energy turnover. A recent study suggested that FD patients may have a marginally reduced cancer rate but possibly increased rates of melanoma, urological malignancies and meningiomas [20]. These data might be supported by our proteome analysis, identifying some proteins involved in cancer. For example, Fibronectin (FN1) is reported to promote melanoma metastasis by inhibiting apoptosis and regulating epithelial-mesenchymal transition (EMT) [21] and is also associated with bladder cancer progression [22]. In addition, loss of fumarate hydratase (FH) is associated with an EMT signature via the suppression of miR-200 in patients suffering from renal cancer [23]. However, these types of connections need to be analyzed in future studies.

### 3.3. Rescue and Normalization of Protein Expression

Current gene therapy aims to either overexpress wild-type AGAL in certain cells for cross correction, or specifically targets FD-relevant tissues. Our proteome analyses confirmed that an (inducible) overexpression of AGAL in FD podocytes results in increased AGAL expression and activities. However, our proteome analyses did only show slight beneficial effects in the rescued podocytes without complete normalization of protein expression patterns compared to the wild-type. This might be due to several reasons. First, dysregulated pathways (i.e., inflammation, etc.) due to deficient AGAL activity are too severely affected by cellular accumulation of Gb_3_ and its derivatives. Therefore, a subsequent induction of functional AGAL expression cannot normalize protein levels in the given time. In this respect, additional treatments (anti-inflammatory) or at least longer induction of AGAL expression phases might be necessary. Second, the massive amount of overexpressed AGAL might mask beneficial effects or caused additional dysregulations. The high synthesis rate requires a lot of energy and might lead to increase metabolic stress and also ER stress in rescued cells. To overcome this potential pitfall, the endogenous *GLA* promotor might be more feasible to drive AGAL expression. An effect of puromycin or lentiviral-transfection can be excluded since all three podocyte cell lines (wild-type, KO and rescue) were treated equally. However, our data are important with respect to the current gene therapy approaches, and future studies should analyze these effects in gene therapy-treated patients.

### 3.4. Validation of Dysregulated Pathways and Functions in Other AGAL-Deficient Cell Lines

FD is a multisystemic disease, affecting numerous organs and cell types. Thus, we analyzed representative key proteins and functions in AGAL-deficient endothelial cells, epithelial kidney cells and patient-specific fibroblast-like cells. Our data demonstrate comparable dysregulated protein expressions in all analyzed cell lines as well as dysregulated cellular functions such as replication and viability. Importantly, we were also able to demonstrate increased ASAH1 expression in a FD kidney biopsy specimen. These data indicate that the observed dysregulated pathways in podocytes are not unique to these cells, but could be transferred to other cell types, underlining the fact that FD is a multisystemic disease with a heterologous clinical phenotype.

### 3.5. Limitations

Our study is the first to show a profound dysregulated protein expression in an adequate AGAL-deficient podocyte cell model. However, future studies are now warranted to further analyze the impact of the dysregulated proteins on cellular function and to transfer our findings from a cell model to the whole organ.

## 4. Materials and Methods

### 4.1. Cell Culture Maintenance

Human immortalized podocytes, HEK293T, EA.hy926 and patient-derived urinary fibroblast cells were cultivated as previously described [10,11,24,25]. Podocytes were cultured at the permissive temperature of 33 °C and transfected using Lipofectamin 2000 (Thermo Fisher Scientific, Darmstadt, Germany) [26].

### 4.2. Proteome Analysis and Statistics

We used MS/MS spectral analysis to identified proteins and label-free quantification (LFQ) to perform statistical analysis with homemade python script. First, LFQ expression was log2 transformed and data were filtered. To keep the graphs compact, we used the corresponding gene name (according to HUGO) instead of the protein name or Ensembl-ID. To detect differentially expressed proteins in two groups and to perform statistical analyses, shared and uniquely expressed proteins in both samples were selected. A student’s unpaired, two-tailed t test and the fold change (FC) were conducted for significance analysis. The FC were calculated by subtraction of the mean values of two groups. FC values of >1 or <−1 (*p* value = 0.05) were defined as significantly regulated. Hierarchical clustering was performed using Euclidian distance measurement technique with standardized data (z-Score).

For gene set-enrichment analysis (GSEA), proteins including at least 2 of 3 values for each triplicate for all groups where selected (1339 proteins) and imputation of the missing values (zero LFQ values) were performed using the random forest algorithm of the python library scikit-learn (RandomForestRegressor). To identify enriched proteome terms, a gene ontology (GO) analysis of significantly regulated genes/proteins (FC > 1 or FC < −1 and *p* value <0.01) and gene-set enrichment analysis (GSEA) were performed using the python library GSEApy with default settings (permutation_num = 1000, method = ‘signal_to_noise’) [27,28,29]. The gene ontology analysis was based on the following webpage (https://maayanlab.cloud/Enrichr/ accessed on 18.10.2021). The KEGG library was used to interpret protein signatures, *p* value was calculated using right-sided hypergeometric tests and the Benjamini–Hochberg correction (fdr) for multiple testing. A term was defined as significantly enriched if the *p* value was <0.05 and fdr < 0.25. The normalized enrichment scores (nes) of GSEA were used to interpret the pairwise comparison of the groups.

### 4.3. CRISPR/Cas9-Mediated GLA Knockout in Podocytes

As reported previously10, the guide (g) RNA was designed using a free online tool previously offered by the Zhang Lab (MIT; http://crispr.mit.edu assessed on 10.10.2018). A single gRNA (5′-ATGCCCAGACCTTTGCTGACTGG-3′; on-target locus: chromosome X: +101401721; quality score: 65; no predicted off-target sites in genes) recognizing the protospacer adjacent motif at position c.481 in exon 3 within the GLA coding sequence was inserted via BbsI in the plasmid pSpCas9(BB)−2A-Puro (PX459) V2.0, which was a gift from Feng Zhang (addgene plasmid no. 62988) [30]. The plasmid was transfected with Lipofectamine 2000 (Thermo Fisher Scientific, Darmstadt, Germany) into podocytes to generate an undirected insertion or deletion via double string break and non-homologous end joining. After transfection and single-cell separation, several clones were analyzed revealing different GLA mutations, some of which showing a complete loss of enzymatic activity and no detectable protein by Western blot analysis due to protein truncations.

To generate an inducible AGAL wild-type rescue, cDNAs from human wild-type AGAL was inserted into the pINDUCER21_puro plasmid [31] and subsequently integrated within the genome for doxycycline-induced gene expression. The integrity of the construct was confirmed by direct sequencing. The monoclonal stable podocyte cell lines allowing doxycycline-dependent expression of wild-type and mutant AGAL were generated by lentiviral gene transfer as previously described [31].

### 4.4. AGAL Expression and Sample Preparation for Proteome Analyses

Wild-type, AGAL-deficient, and AGAL-overexpressing cells were grown until confluence and treated for 7 days with doxycycline (0.2 µg/mL). Media with doxycycline was renewed after 2, 4, and 6 days. At day 7, cells were harvested and lysed in 100 µL lysis buffer (6 M urea, 2 M thiourea, 50 mM Tris, 1 complete tablet (Roche, Mannheim, Germany) per 10 mL; pH 8.0) and stored at −80 °C until proteome analysis.

### 4.5. Western Blot Analyses

For Western blot analyses, 10 µg samples were blotted onto PVDF membranes. After blocking overnight in Tris buffered saline with 5% milk powder, detection was per-formed using either an anti-AGAL antibody (ab168341, Abcam, Cambridge, UK), anti-ASAH1 antibody (PA5-52150, Thermo Fisher Scientific, Darmstadt, Germany) or anti-RAB11B antibody (ab175925, Abcam, Cambridge, UK) combined with a secondary horseradish-peroxidase-labeled goat anti-rabbit IgG antibody (12-348, Sigma-Aldrich, St. Louis, MO, USA).

### 4.6. AGAL Enzyme Activity Assay

Cells were lysed in passive lysis buffer (Promega, Mannheim, Germany). For normalization, the protein yield was quantified using BCA reagent (Thermo Fisher Scientific, Darmstadt, Germany). AGAL activity was measured using 4-methylumbelliferone-α-D-galactopyranoside (Biosynth, Staad, Switzerland) as described elsewhere [32]. N-Acetylgalactosamine (Carbosynth, Compton, UK) was used as inhibitor for α-galactosidase B activity [33].

### 4.7. Proliferation Rate and Lysosome Staining

To determine the proliferation rate of cell lines, the cell numbers per milliliter after incubation were quantified. EA.hy926 cells were seeded at densities range from 0.75 × 10^5^ to 2.5 × 10^5^ cells/mL and HEK293T cells from 2.25 × 10^5^ to 4 × 10^5^ cells/mL. Cells were seeded in 48-well plates for 48h (HEK293T) or 72 h (EA.hy926) at 37 °C. After the indicated time, cells were washed with PBS and dissolved by adding 125 µL of Trypsin-EDTA (0.05%) in DPBS (1x) (Capricorn scientific; Ebsdorfergrund, Germany; TRY-1B). After stopping and removing trypsin, cells were resuspended in 250 µL medium and counted using a Neubauer chamber. Cell viability assays (XTT; A2240, AppliChem, Darmstadt, Germany) were performed according to manufacturer’s instructions. Lysosomal staining with Lysotracker (L7528; Thermo Fisher Scientific, Darmstadt, Germany) was performed according to manufacturer’s instructions.

### 4.8. Immunohistochemistry in Kidney Biopsy Specimen

ASAH1 immunohistochemistry was performed on 3 μm thick, formalin fixed and paraffin embedded tissue sections using a primary rabbit polyclonal anti-ASAH1 antibody (PA5-52150, Thermo Fisher Scientific, Darmstadt, Germany), 1:500 on an automated staining system (Ventana BenchMark Ultra, Roche, Mannheim, Germany). Negative controls were obtained by omission of the primary antibody. Slides were pretreated with Cell Conditioning Solution CC1 (Ventana Medical Systems/Roche, Basel, Switzerland) for 32 min. For visualization the OptiView DAB IHC Detection Kit (VENTANA/Roche, Mannheim, Germany) was used.

Representative images were acquired using the Diskus imaging software (Hilgers Technisches Büro, Königswinter, Germany).

## 5. Conclusions

We conclude that a loss of AGAL function results in profound changes of cellular pathways. These changes are not unique to a specialized cell type but are ubiquitous to various other cell types, explaining the heterogeneity of FD phenotypes. Due to the profound changes, current approved FD-specific therapy might not be sufficient to reverse all dysregulated pathways.

## Data Availability

All data and material are present within the main manuscript and supplemental materials.

## Supplementary Materials

The following are available online at https://www.mdpi.com/article/10.3390/ijms222111339/s1.
